# METTL3 Promotes Activation and Inflammation of FLSs Through the NF-κB Signaling Pathway in Rheumatoid Arthritis

**DOI:** 10.3389/fmed.2021.607585

**Published:** 2021-07-06

**Authors:** Wen Shi, Yan Zheng, Shuai Luo, Xiaofeng Li, Yilong Zhang, Xiaoming Meng, Cheng Huang, Jun Li

**Affiliations:** ^1^Inflammation and Immune Mediated Diseases Laboratory of Anhui Province, Hefei, China; ^2^Anhui Institute of Innovative Drugs, School of Pharmacy, Anhui Medical University, Hefei, China; ^3^The Key Laboratory of Anti-inflammatory and Immune Medicines, Ministry of Education, Hefei, China

**Keywords:** rheumatoid arthritis, METTL3, FLSs, inflammatory response, proliferation, invasion, migration

## Abstract

Rheumatoid arthritis (RA), a common autoimmune disease, is extremely damaging to human health. Fibroblast-like synoviocytes (FLSs) have a vital role in the occurrence and development of RA. Methyltransferase-like 3 (METTL3), which is a crucial component of the *N*^6^-methyladenosine (m^6^A) methyltransferase complex, is involved in the progression of many diseases. In this study, we explored the role of METTL3 in the inflammatory response and proliferation, invasion, and migration of FLSs. We used human RA synovial tissues and the adjuvant-induced arthritis (AIA) animal model of RA. Experimental results revealed that METTL3 expression was significantly upregulated in human RA synovial tissues and in the rat AIA model. METTL3 knockdown suppressed interleukin (IL)-6, matrix metalloproteinase (MMP)-3, and MMP-9 levels in human RA-FLSs and rat AIA-FLSs. In contrast, they were increased by METTL3 overexpression. Additionally, we found that, in FLSs, METTL3 may activate the nuclear factor (NF)-κB signaling pathway. The experimental results showed that METTL3 may promote FLS activation and inflammatory response *via* the NF-κB signaling pathway.

## Introduction

Rheumatoid arthritis (RA), a common autoimmune dysfunction disease, is mainly a joint disease, but it also involves multiple other systems (1). As a chronic inflammatory disorder, it involves synovial hypertrophy, severe joint damage, and loss of function (2, 3). It often develops in middle age and is more common among women than in men (4). RA patients have a greater risk of developing malignant tumors than the general population (5). RA is extremely damaging to human health, and the exact pathogenesis is not fully understood (6).

Increasing research suggests that fibroblast-like synoviocytes (FLSs) have a vital role in the pathogenesis. As the main cells involved in the pathogenesis, they cause synovial hyperplasia, pannus formation, and cartilage and bone tissue erosion, eventually destroying nearby cartilage and bone tissue (2, 7). The hyperproliferating FLSs release a large number of pro-inflammatory indicators, such as interleukin (IL)-6, IL-1β, and tumor necrosis factor (TNF)-α, causing abnormal inflammation in the synovial membrane (8, 9). What is more, the activated FLSs release matrix metalloproteinases (MMPs), which promote FLS migration and invasion (10, 11). Therefore, reducing the activation and inflammatory response of FLSs is a promising therapeutic strategy for the treatment of RA (8, 12, 13).

RNA *N*^6^-methyladenosine (m^6^A) represents a particularly common RNA modification (14). In recent years, research has found that this modification plays crucial roles in cancer, autoimmunity, and inflammation (15, 16). Methyltransferase-like 3 (METTL3) was not only the first identified constituent of methyltransferase but also the core component of the methyltransferase complex, which involves METTL3, METTL14, and WTAP (17, 18). It has been proven that METTL3-mediated m^6^A methylation has tissue and cell specificity. METTL3 affects the methylation of specific target genes, thereby regulating embryo development, endothelial-to-hematopoietic transformation, and other important bioprocesses (19, 20). In RA patients, METTL3 is upregulated both in peripheral blood mononuclear cells and in monocytes (21). Nevertheless, the role of METTL3 in RA-FLSs is unknown.

It is found that METTL3 is believed to activate the nuclear factor (NF)-κB signaling pathway (21), leading to tumor progression, migration, and invasion. In bladder cancer, METTL3 promotes cancer development *via* the AFF4/NF-κB/MYC signaling network (22). Additionally, METTL3 regulates osteogenic differentiation *via* the NF-κB signaling pathway (23). Moreover, the NF-κB signaling pathway is sensitized in RA-FLSs and is very important in many ways, inducing inflammatory cytokine secretion and MMP production in FLSs (24–26).

We speculated that it would be of great significance to explore the link between METTL3 and RA. In this study, we aimed to explore the effects of altering METTL3 expression on pro-inflammatory cytokine production and FLS activation.

## Materials and Methods

### Collection of Human Synovial Tissue

We received the synovial tissue from developing RA patients (*n* = 5, aged 40–70 years) who were undergoing a synovectomy or joint replacement and who had been diagnosed on the basis of the rheumatology criteria revised by the American College in 1987. Meanwhile, the control group involved osteoarthritis (OA) patients (*n* = 3, aged 40–70 years). The samples were obtained at the First Affiliated Hospital of Anhui Medical University. All patients involved in this experiment had to submit informed consent. The study plan was given permission by the Anhui Medical University ethics board. At the same time, tissue specimen collection was carried out according to the guidelines of the institution. All participants provided written informed consent.

### Materials and Reagents

Rabbit anti-MMP3, anti-MMP9, and anti-METTL3 antibodies were obtained from Abcam (Cambridge, UK). We obtained the rabbit anti-phospho-(p)-p65 antibody, anti-β-actin antibody, anti-vimentin antibody, and anti-p65 antibody from Cell Signaling Technology, Inc. (Danvers, MA, USA). Rabbit anti-proliferating cell nuclear antigen (PCNA) antibody was obtained from Bioss (Beijing, China). The secondary antibody we used was from Zhongshan Biotechnology Corporation (Beijing, China). The cell cycle kits we used were from BestBio (Shanghai, China). The enzyme-linked immunosorbent assay (ELISA) kits for IL-6 were purchased from Elabscience Biotechnology Co., Ltd. METTL3, MMP3, MMP9, PCNA, IL-6, IL-1β, and TNF-α primers were synthesized by Shanghai Sangon Biological and Technological Company (Shanghai, China).

### Adjuvant-Induced Arthritis Animal Model

Sprague–Dawley (SD) rats (female, weighing ~80–120 g) were obtained from the Laboratory Animal Center of Anhui Medical University. To establish the adjuvant-induced arthritis (AIA) model, the rats were given complete Freund's adjuvant (CFA; Chondrex, Inc.) on the left paw of 0.1 ml per 100 g of body weight. Additionally, the rats were injected with normal saline to create the negative control (NC) group. The animals we obtained were divided into two groups. Each group contains six rats. At day 24 after the injection, each rat was killed. Meanwhile, the synovium tissue was isolated from the knee for further experimentation. In order to minimize damage to animals, the animal experiments followed the regulations on the Administration of Experimental Animals issued by China's State Science and Technology Commission. Additionally, the experimental program related to animals has been approved by the Animal Care and Use Committee.

### Histopathology and Immunohistochemical Staining

The synovial tissues (human and rat synovial tissues) we obtained were soaked in 10% paraformaldehyde for 24 h, embedded in paraffin, sectioned (5 μm thick), and stained with hematoxylin and eosin (H&E) staining. Meanwhile, the paraffin-embedded tissues (5 μm thick) underwent immunohistochemical staining to detect METTL3, which involved desiccation, dewaxing, and hydration. After that, antigen retrieval and goat serum blocking were performed. Then, the tissues were incubated with an anti-METTL3 antibody overnight and treated with the secondary antibody. Thereafter, the sections were observed using a microscope (Olympus, Tokyo, Japan) at ×20 magnification to assess the changes in vasospasm, inflammatory cell infiltration, and synovial hyperplasia.

### Paw Swelling Score and Rat Arthritis Scores

Firstly, two groups of rats were put in cages individually. Paw volume (relative to the paw volume before CFA/saline injection on day 1) was assessed every other day from day 12 to 20. Additionally, on the same days, the rat arthritis score was determined based on previously described criteria (27).

### Cytokine Assay by ELISA

ELISA kits (Elabscience Biotechnology Co. Ltd) were used to assess IL-6 expression in the TNF-α-treated FLS supernatants following the manufacturer's instructions.

### Cell Culture and Treatment

The synovial tissues were used to derived FLSs. The synovial tissues were cut into pieces and placed in cell culture flasks immediately after collection. The culture system was dominated by a high-glucose Dulbecco's modified Eagle's medium (DMEM; HyClone, USA) with 20% (vol/vol) fetal bovine serum (FBS; Gibco, USA) and 1% (vol/vol) penicillin–streptomycin solution (Beyotime, China). The cells were then placed in an incubator at a temperature of 37°C with 5% CO_2_. Cells from the third to the fifth generation were used in the subsequent experiments. Vimentin, which was detected using an immunofluorescence assay, was used to identify the FLSs. In the following cell experiments, to activate the FLSs, they were treated with TNF-α at a working concentration of 10 ng/ml.

### Cell Viability Assay

To analyze the viability of FLSs, 3-(4,5-dimethylthiazol-2-yl)-2,5-diphenyltetrazolium bromide (MTT) assays were used. The FLSs were placed in 96-well plates at an amount of 6,000 cells per well. The cells were cultured for 24 h after transfection. Thereafter, 20 μl/well MTT working solution (Sigma, USA) was added. After 4 h, the liquid was aspirated and 200 μl dimethylsulfoxide (DMSO) was added to each well. Lastly, the absorbance at 490 nm was measured by a microplate reader (Synergy HTX, Biotek, Winowski, VT, USA).

### Cell Cycle Assay

Firstly, the FLSs were grown in six-well plates and treated with METTL3 small interfering RNA (siRNA) and METTL3 overexpression plasmid. After 48 h, the FLSs were trypsinized, fixed in 70% ethanol, and placed in 4°C stay overnight. FLSs were centrifuged for 10 min, the speed set to 1,000 × g, and resuspended in PBS. After that, the cells were treated with 20 μl RNase A at a temperature of 37°C for 30 min. Next, 400 μl propidium iodide staining buffer was added to each tube, which was then placed in darkness at 4°C for 30 min. Fluorescence-activated cell sorting (FACS) was then conducted using Beckman Coulter.

### Small RNAi Transfection

Small interfering RNA targeting METTL3 of humans and rats was obtained from GenePharma Corporation (Shanghai, China) and Bioogenetech Corporation (Shanghai, China), respectively. The METTL3-RNAi (rat) sense strand is 5′-CCGGUUUAAGCACAGGUAUTT-3′ and the antisense strand is 5′-AUACCUGUGCUUAAACCGGGC-3′. The METTL3-RNAi (human) sense strand is 5′-GCUCAACAUACCCGUACUATT-3′ and the antisense strand is 5′-UAGUACGGGUAUGUUGAGCTT-3′. Before transfection, the cells were seeded into six-well plates. The transfection was conducted using jetPRIME transfection reagent (Polyplus-transfection, Illkirch, France) in accordance with the experimental instructions. After 4–6 h, the culture medium was replaced with fresh medium and then TNF-α (10 ng/ml) was added for another 48 h. The cells were then subjected to a series of assays.

### Plasmid Construction and Transfection

The overexpression plasmid for human was METTL3-pcDNA3.1, obtained from GenePharma Corporation (Shanghai, China), and the overexpression plasmid for rat was METTL3-PEX, received from Bioogenetech Corporation (Shanghai, China). Before transfection, the cells were cultured in six-well plates. The transfection was conducted using the jetPRIME transfection reagent (Polyplus-transfection, Illkirch, France) in accordance with the experimental instructions. After 4–6 h of transfection, we used fresh medium to replace the culture medium and then incubated with TNF-α at a concentration of 10 ng/ml for another 48 h. After that, we carried out the subsequent series of experiments.

### Wound Healing Assay

The FLSs (1.0 × 10^5^/well) were placed in six-well plates containing DMEM with 20% FBS for 24 h. To create a wound in the cell monolayer in each well, the monolayer was manually scraped with a 10-μl pipette tip and then washed with PBS to remove the cells that were no longer attached to the plate after being scraped. Afterwards, the cells were treated with small interfering RNA or the overexpression plasmid in serum-free DMEM for 24 h. Thereafter, the cells were fixed with 4% paraformaldehyde, stained with 0.1% crystal violet, and were then observed using an Olympus BX-51 microscope.

### Transwell Invasion Assay

Special 24-well-plates were used for the Transwell invasion assay. The 24-well plates have chamber inserts with an 8-μm pore size (#3422, Corning Inc.), following the manufacturer's instructions. Firstly, we extracted and diluted the Matrigel in a scale of 1:10 and added it to the upper chamber. After 4 h, the cell suspension (RA-FLSs or AIA-FLSs) with 1% FBS was placed in the upper chamber. The normal culture medium was placed in the lower chamber. Twenty-four hours later, 4% paraformaldehyde was used to fix the migrated cells and 0.1% crystal violet was used for staining; the cells were observed with an Olympus BX-51 microscope.

### Western Blotting

One percent protease inhibitor cocktail (Beyotime, China) was added to the radioimmunoprecipitation assay (RIPA) lysis buffer in advance. Then, we used it to lyse the cultured cells and synovial tissues. The BCA protein assay kit (Beyotime, China) was used to assess the extracted protein concentrations. The protein samples were separated with 10% SDS–polyacrylamide gel and then transferred onto polyvinyl difluoride membranes (Millipore, USA). Thereafter, the membranes were blocked at room temperature. Two hours later, the membranes were incubated at 4°C with a specific primary antibody (anti-MMP-3, anti-MMP-9, and anti-METTL3 were used at a dilution ratio of 1:500; anti-β-actin, anti-p65, and anti-p-p-P65 were diluted at a ratio of 1:1,000) overnight. They were then washed three times with Tris-buffered saline with Tween 20. The secondary antibody was used at a dilution ratio of 1:5,000 to incubate the membranes for 1 h at room temperature. In the end, enhanced chemiluminescence kit (Thermo Scientific, USA) was used to show the protein bands. The protein expression was quantified using ImageJ software.

### Real-Time Quantitative PCR

To detect the messenger RNA (mRNA) levels of METTL3, IL-6, MMP-3, MMP-9, PCNA, and β-actin (the primer sequences are shown in Tables 1, 2), total RNA was first extracted with a TRIZOL reagent (Invitrogen, USA). The concentration was quantified by a NanoDrop 2000 spectrophotometer (Thermo Scientific, USA). The 5× PrimeScript RT Master Mix (TAKARA, Japan) was used to obtain complementary DNA (cDNA) by reverse transcription. We selected the TB Green qPCR Premix Ex Taq II (TAKARA, Japan) to prepare the reaction mixture on the basis of the experimental instructions. The reaction was performed in a Pikoreal 96 real-time PCR system (Thermo Scientific, USA) to detect the mRNA levels using the following conditions: 95°C for 10 min and then 40 cycles at 95°C for 15 s and 60°C for 60 s. Relative expression was calculated using the 2^−ΔΔCt^ method, with β-actin mRNA expression used as the internal reference.

**Table 1:** Primer sequences used in real-time quantitative PCR for rat genes.

**Gene**	**Primer sequence**
METTL3	Forward: 5′-CTGGCACCCGAAAGATTGAG-3′
	Reverse: 5′-CATCTGGGTCCAGAAGGTGT-3′
IL-6	Forward: 5′-ACTGACAACACCGGAAGCCA-3′
	Reverse: 5′-GGCCGGCAGTGAGAAACTTG-3′
MMP-3	Forward: 5′-ACCTATTCCTGGTTGCTGCT-3′
	Reverse: 5′-CAGGTCTGTGGAGGACTTGT-3′
MMP-9	Forward: 5′-AGTGCCCTTGAACTAAGGCT-3′
	Reverse: 5′-GCCTCCACTCCTTCCTAGTC-3′
PCNA	Forward: 5′-GAAGAAGGTGCTGGAGGC-3′
	Reverse: 5′-TTGGACATGCTGGTGAGG-3′
β-actin	Forward: 5′-CCCATCTATGAGGGTTACGC-3′
	Reverse: 5′-TTTAATGTCACGCACGATTTC-3′

**Table 2:** Primer sequences used in real-time quantitative PCR for human genes.

**Gene**	**Primer sequence**
METTL3	Forward: 5′-GCCTTCTGAACCAACAGTCC-3′
	Reverse: 5′-CTGGCTTTCATGCACTCCTC-3′
IL-6	Forward: 5′-AGACAGCCACTCACCTCTTC-3′
	Reverse: 5′-AGTGCCTCTTTGCTGCTTTC-3′
MMP-3	Forward: 5′-ACTCGAGTCACACTCAAGGG-3′
	Reverse: 5′-ACAAGGTGCAAGCTAAGCAG-3′
MMP-9	Forward: 5′-GACAAGCTCTTCGGCTTCTG-3′
	Reverse: 5′-CAAAGTTCGAGGTGGTAGCG-3′
PCNA	Forward: 5′-GGCACTCAAGGACCTCAT-3′
	Reverse: 5′-TGTCGAAGCCCTCAGACC-3′
β-actin	Forward: 5′-CCCTGGAGAAGAGCTACGAG-3′
	Reverse: 5′-GGAAGGAAGGCTGGAAGAGT-3′

### Statistical Analysis

All of these values are presented as the mean ± SD, and the results were acquired from at least three experiments. We used paired *t* test for the difference comparison within intra-group. ANOVA was used to compare the differences among groups. All data were analyzed using SPSS 23.0 software. *P* < 0.05 was defined as statistically significant.

## Results

### METTL3 Was Obviously Upregulated in RA-FLSs

**Figure 1:**
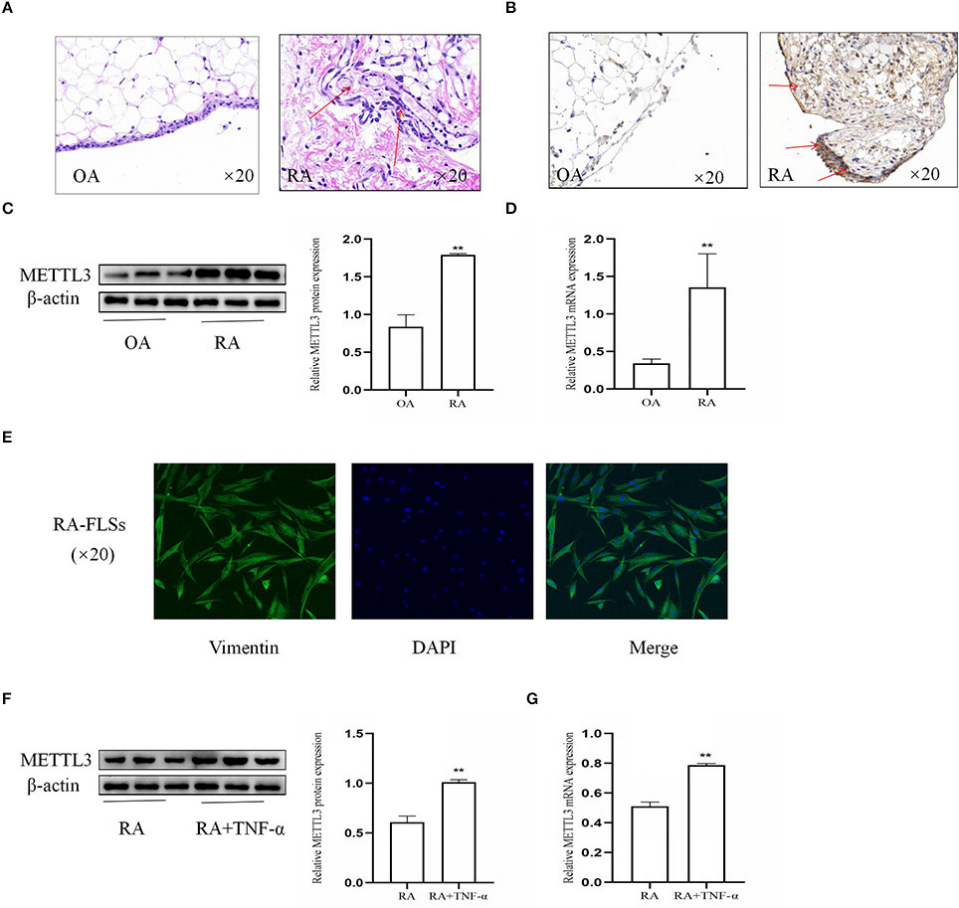
Methyltransferase-like 3 (METTL3) was obviously upregulated in rheumatoid arthritis fibroblast-like synoviocytes (RA-FLSs). **(A)** H&E staining was used to analyze synovial tissues (shown as *red arrows*) from RA and osteoarthritis (OA) patients (original magnification, ×20). **(B)** METTL3 expression in synovial tissues (shown as *red arrows*), as detected by immunohistochemical staining (original magnification, ×20). **(C)** Western blotting was used to detect METTL3 protein level in synovial tissues. **(D)** METTL3 mRNA levels in OA and RA synovial tissues, as detected by real-time quantitative PCR (qPCR). **(E)** Vimentin expression, as detected by immunofluorescence (original magnification, ×20); the extracted cells were verified to be RA-FLSs. **(F)** METTL3 protein levels in RA-FLSs after treatment with TNF-α, as detected by Western blotting. **(G)** METTL3 mRNA levels in RA-FLSs after TNF-α incubation, as detected by qPCR. All of these values are presented as the mean ± SD. *OA*, osteoarthritis. **P < 0.01 vs. the OA control group.

To explore the importance of METTL3 in RA, the synovial tissues from patients with OA and RA were used. Firstly, the histopathologic analysis (Figure 1A) showed that, in the RA synovial tissues compared to the OA synovial tissues, the inflammatory characteristics were more obvious, with clearly increased inflammatory cell infiltration, pannus formation, and enhanced synovial hyperplasia. Additionally, immunohistochemical analysis (Figure 1B) of the synovial tissues indicated that METTL3 was increased in RA compared to OA. Western blotting results (Figure 1C) showed that METTL3 expression was upregulated in RA synovial tissues. The real-time quantitative PCR (qPCR) results (Figure 1D) further revealed that METTL3 expression was increased in the RA synovial tissues. The expression of vimentin, which was used to identify FLSs (28), showed that the cells we extracted from the RA synovial tissues were FLSs (Figure 1E). The Western blotting and qPCR results (Figures 1F,G) confirmed that METTL3 obviously increased in RA-FLS with TNF-α (10 ng/ml) treatment. In conclusion, the results showed that METTL3 is clearly upregulated in both RA synovial tissues and FLSs.

### METTL3 Expression Was Significantly Upregulated in AIA

**Figure 2:**
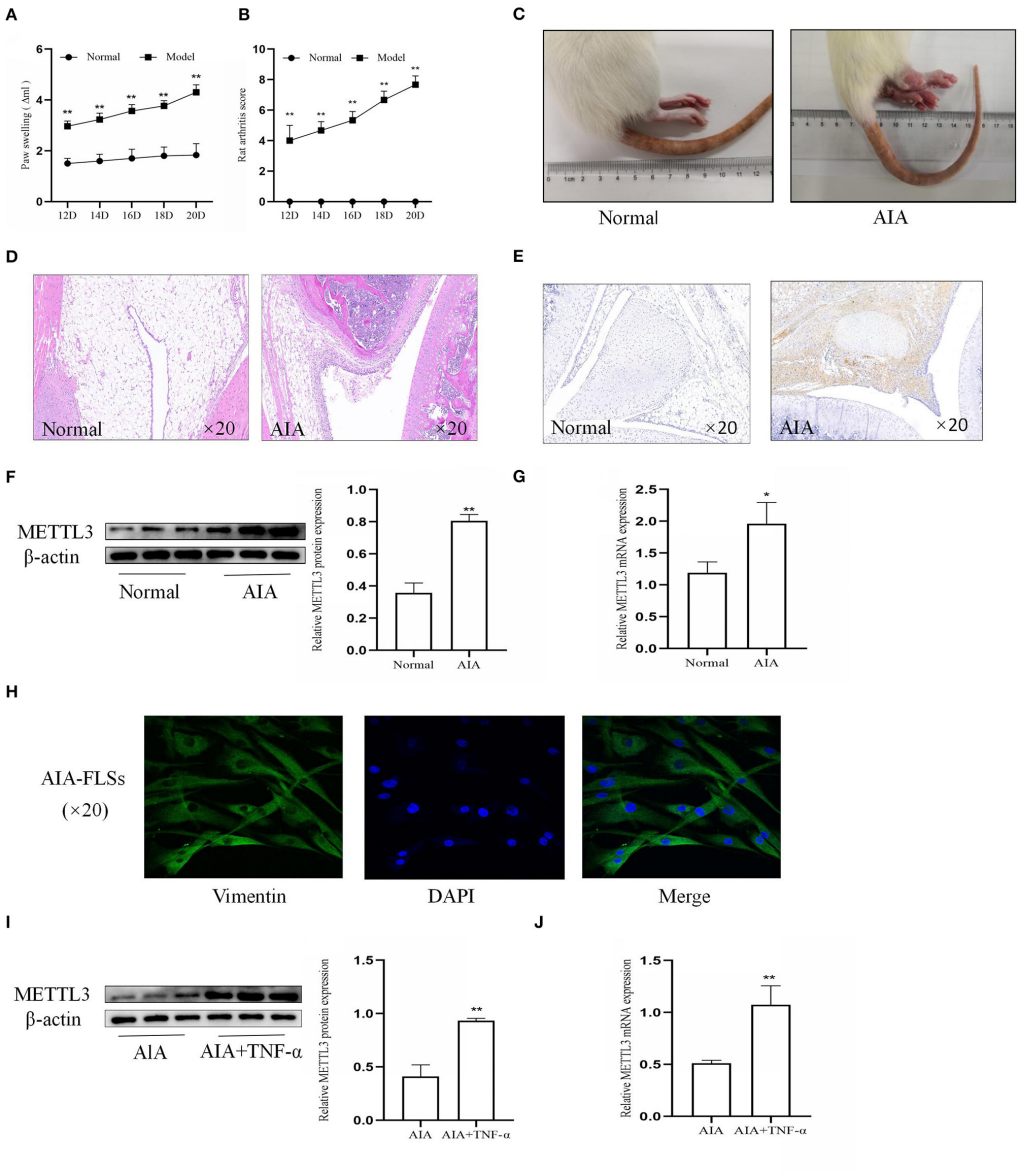
Methyltransferase-like 3 (METTL3) expression was significantly upregulated in adjuvant-induced arthritis (AIA). **(A)** Ankle joint swelling based on Plethysmometer measurements. **(B)** Arthritis scores were extremely increased in the AIA model group. **(C)** Representative photographs of morphology in the normal group and the AIA model on day 24 after complete Freund's adjuvant (CFA) induction. Typical photos were taken. **(D)** H&E staining of synovial tissues in the normal group and AIA group (original magnification, ×20). **(E)** METTL3 expression in the normal and AIA groups, as detected by immunohistochemical staining (original magnification, ×20). **(F)** Western blotting was used to measure METTL3 protein levels in normal and AIA synovial tissues. **(G)** METTL3 mRNA level was detected by real-time quantitative PCR (qPCR) in normal and AIA synovial tissues. **(H)** Cell identification, as detected by immunofluorescence (original magnification, ×20), vimentin expression, and morphology, showed that the extracted cells were AIA fibroblast-like synoviocytes (FLSs). **(I)** METTL3 protein levels in AIA-FLSs after treatment with TNF-α, as detected by Western blotting. **(J)** qPCR was used to measure METTL3 mRNA levels in AIA-FLSs after treatment with TNF-α. There were six rats in the normal group as well as in the model group. All of these values are presented as the mean ± SD. *NC*, negative control. *P < 0.05, **P < 0.01 vs. the normal group.

To further confirm the effect of METTL3, CFA was injected into the rat paws to establish the AIA model. The rat arthritis score (Figure 2A) and paw swelling score were evaluated (Figure 2B). Representative photographs (Figure 2C) showed that the right paws of the AIA rats tended to be more clearly red and swollen. Histopathological analysis (Figure 2D) indicated that the AIA group had a remarkably increased inflammatory cell infiltration compared to the normal group. These results showed that the AIA model was successfully established. To assess the expression of METTL3 in the normal group and the AIA group, immunohistochemical analysis (Figure 2E), Western blotting (Figure 2F), and qPCR (Figure 2G) were conducted. The results all indicated that compared to the normal group, METTL3 was upregulated in the AIA group. Vimentin level (Figure 2H) showed that the extracted cells were FLSs. The Western blotting (Figure 2I) and the qPCR (Figure 2J) results confirmed that METTL3 was upregulated in AIA-FLSs treated with TNF-α (10 ng/ml). Thus, these results showed that METTL3 expression was observably increased in both AIA synovial tissues and AIA-FLSs.

### METTL3 Silencing Inhibits Inflammatory Cytokine in FLSs

**Figure 3:**
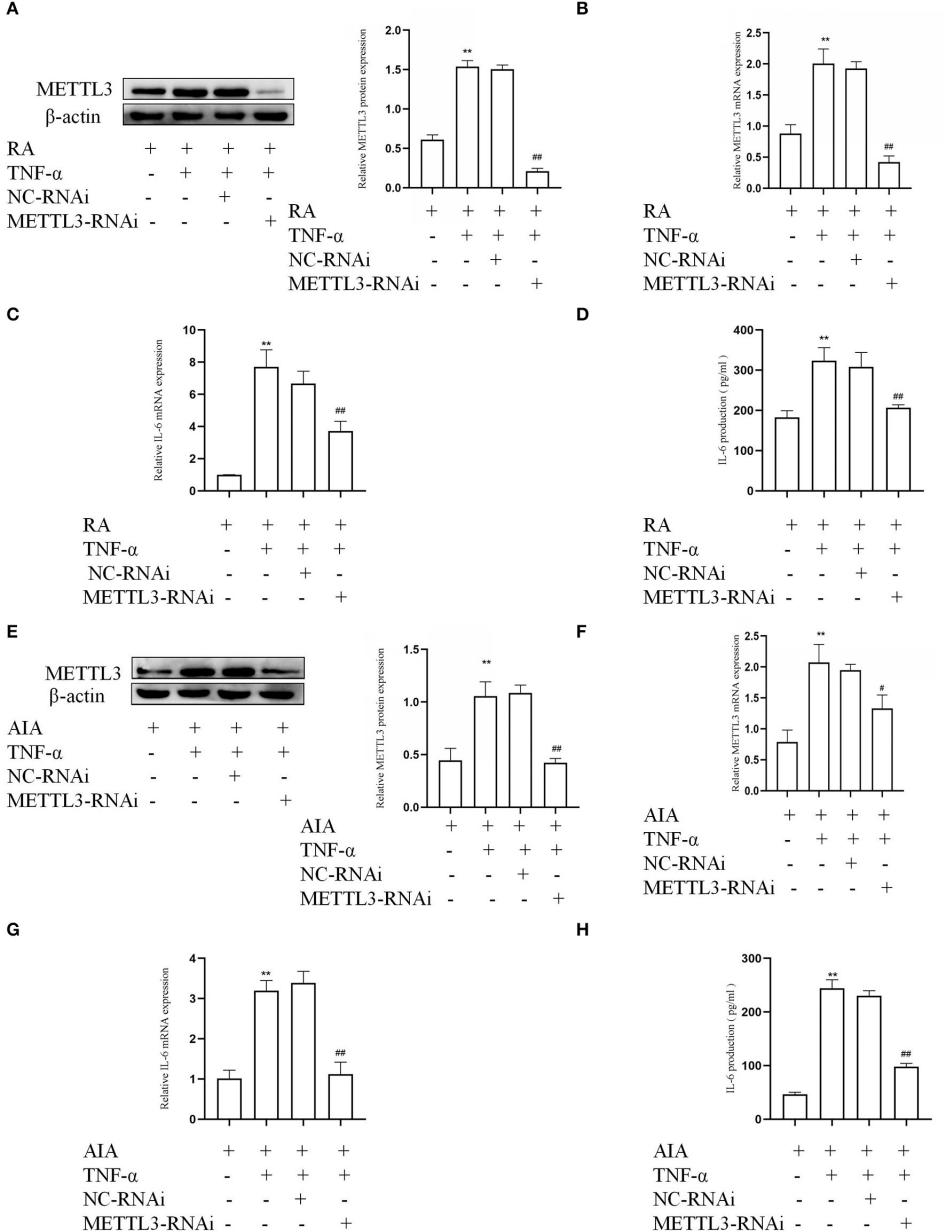
Methyltransferase-like 3 (METTL3) knockdown (using METTL3 siRNA) inhibits inflammatory cytokines in human and rat fibroblast-like synoviocytes (FLSs). **(A)** METTL3 protein levels in TNF-α-treated rheumatoid arthritis (RA)-FLSs after METTL3 knockdown, as detected by Western blotting. **(B)** METTL3 mRNA levels in TNF-α-incubated RA-FLSs after METTL3 silencing, as detected by real-time quantitative PCR (qPCR). **(C)** IL-6 mRNA levels in TNF-α-incubated RA-FLSs with METTL3 knockdown, as detected by qPCR. **(D)** The number of IL-6 in TNF-α-incubated RA-FLS supernatants were analyzed by ELISA. **(E)** Western blotting was used to measure METTL3 protein levels in TNF-α-incubated AIA-FLSs with METTL3 siRNA. **(F)** METTL3 mRNA levels in TNF-α-incubated AIA-FLSs after using METTL3 siRNA, as detected by qPCR. **(G)** IL-6 mRNA levels in TNF-α-incubated AIA-FLSs with METTL3 knockdown, as detected by qPCR. **(H)** ELISA was used to detect the production of IL-6 in TNF-α-incubated AIA-FLS supernatants. All of these values are presented as the mean ± SD. *NC*, negative control. **P < 0.01 vs. the control group. ^#^*P* < 0.05, ^##^*P* < 0.01 vs. the NC group.

To determine whether METTL3 in RA-FLSs were associated with an inflammatory response, the siRNA-targeted METTL3 was used to downregulate the METTL3 level in TNF-α-incubated RA-FLSs. The Western blotting and qPCR results (Figures 3A,B) both indicated that the human-specific METTL3 siRNA obviously decreased the expression of METTL3 in RA-FLSs. We also used rat-specific siRNA-targeted METTL3 to silence the expression in TNF-α-incubated AIA-FLSs. The protein and mRNA amounts of METTL3 (Figures 3E,F) were clearly reduced in the METTL3-siRNA group compared to the NC-siRNA group. Next, to explore the change in inflammatory cytokines in the FLSs, real-time qPCR and ELISA assay were performed. The qPCR results (Figures 3C,G) showed that the IL-6 mRNA levels were downregulated in TNF-α-treated RA-FLSs and AIA-FLSs by METTL3-RNAi transfection. The ELISA results (Figures 3D,H) indicated that IL-6 was obviously decreased in TNF-α-incubated RA-FLSs and AIA-FLSs after METTL3 silencing. The results all confirmed that the inhibition of METTL3 decreased the inflammatory response of RA-FLSs and AIA-FLSs.

### METTL3 Overexpression Along With High Expression of Inflammatory Cytokine

**Figure 4:**
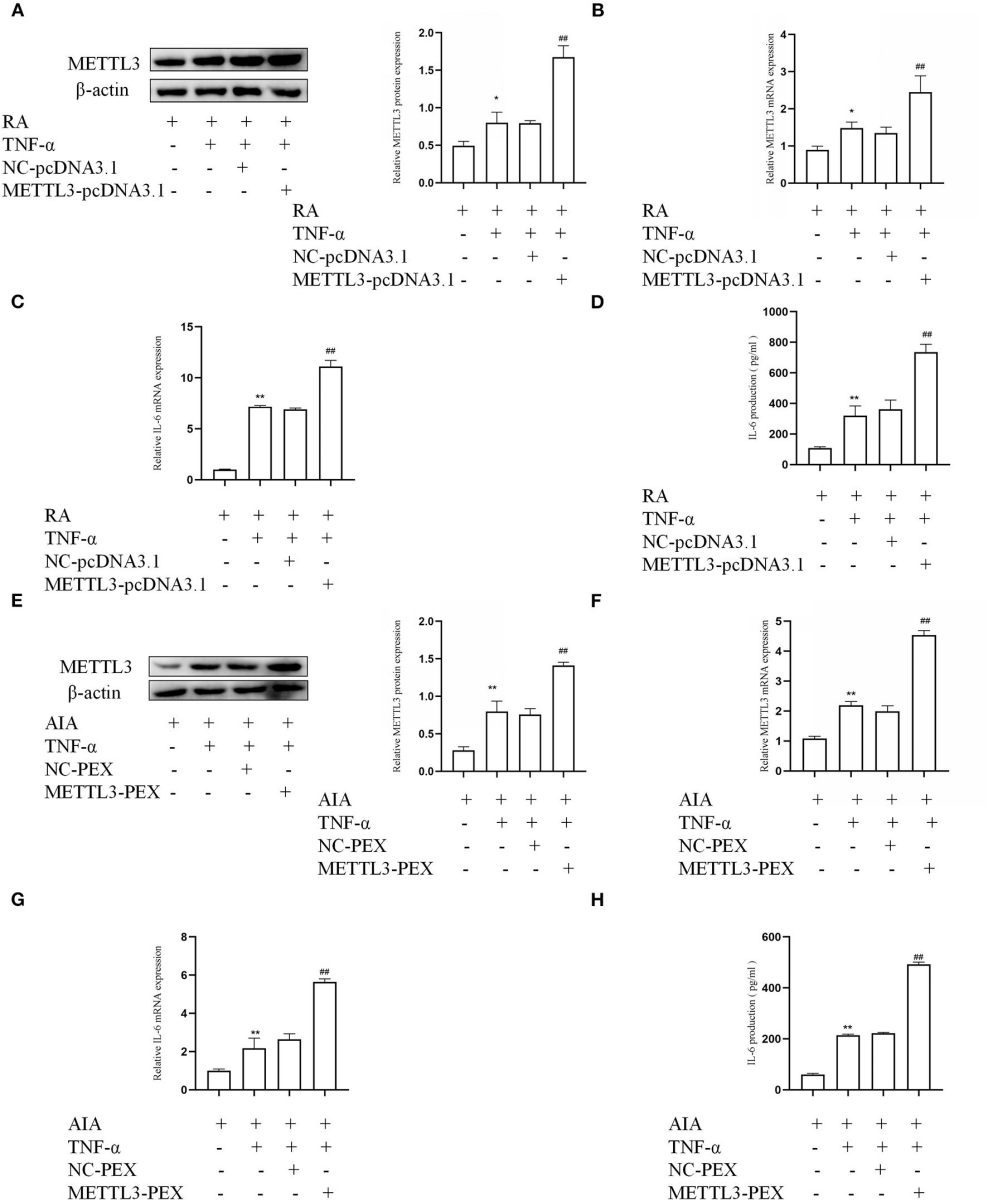
Methyltransferase-like 3 (METTL3) overexpression along with high expression of inflammatory cytokines. **(A)** METTL3 protein levels in TNF-α-incubated rheumatoid arthritis fibroblast-like synoviocytes (RA-FLSs) with METTL3 overexpression, as detected by Western blotting. **(B)** METTL3 mRNA levels in TNF-α-incubated RA-FLSs after overexpressing METTL3, as detected by real-time quantitative PCR (qPCR). **(C)** IL-6 mRNA levels in TNF-α-incubated RA-FLSs after overexpressing METTL3, as detected by qPCR. **(D)** The number of IL-6 in TNF-α-incubated RA-FLS supernatants were analyzed by ELISA. **(E)** METTL3 protein level was measured by Western blotting in TNF-α-incubated AIA-FLSs after METTL3 overexpression. **(F)** METTL3 mRNA level was detected by qPCR in TNF-α-incubated AIA-FLSs after using METTL3-PEX. **(G)** IL-6 mRNA levels were detected by qPCR in TNF-α-incubated AIA-FLSs with overexpressed METTL3. **(H)** The number of IL-6 in TNF-α-incubated AIA-FLS supernatants were measured by ELISA after transfection. All of these values are presented as the mean ± SD. *NC*, negative control. *P < 0.05, **P < 0.01 vs. the control group. ^##^*P* < 0.01 vs. the NC group.

To provide more evidence that METTL3 is associated with an inflammatory response in FLSs, overexpression plasmids were used in RA-FLSs and AIA-FLSs, respectively. The Western blotting (Figure 4A) and qPCR (Figure 4B) results indicated that, in TNF-α-incubated RA-FLSs, compared to the NC group, the expression of METTL3 was obviously increased in the METTL3-pcDNA3.1 group. What is more, the qPCR results (Figure 4C) showed that the pro-inflammatory cytokine mRNA levels were clearly increased by METTL3 overexpression in RA-FLSs with TNF-α treatment. The ELISA results (Figure 4D) also showed that the IL-6 protein levels were upregulated by transfection with METTL3-pcDNA3.1. Moreover, the Western blotting (Figure 4E) and qPCR (Figure 4F) results showed that, compared to the NC group, METTL3 expression was increased in AIA-FLSs transfected with METTL3-PEX. And the qPCR (Figure 4G) and ELISA (Figure 4H) results indicated that the IL-6 levels were obviously upregulated in TNF-α-incubated AIA-FLSs after METTL3-PEX transfection. These results confirmed that METTL3 overexpression increased the inflammatory response of RA-FLSs and AIA-FLSs.

### METTL3 Silencing Inhibits the Cell Proliferation in FLSs

**Figure 5:**
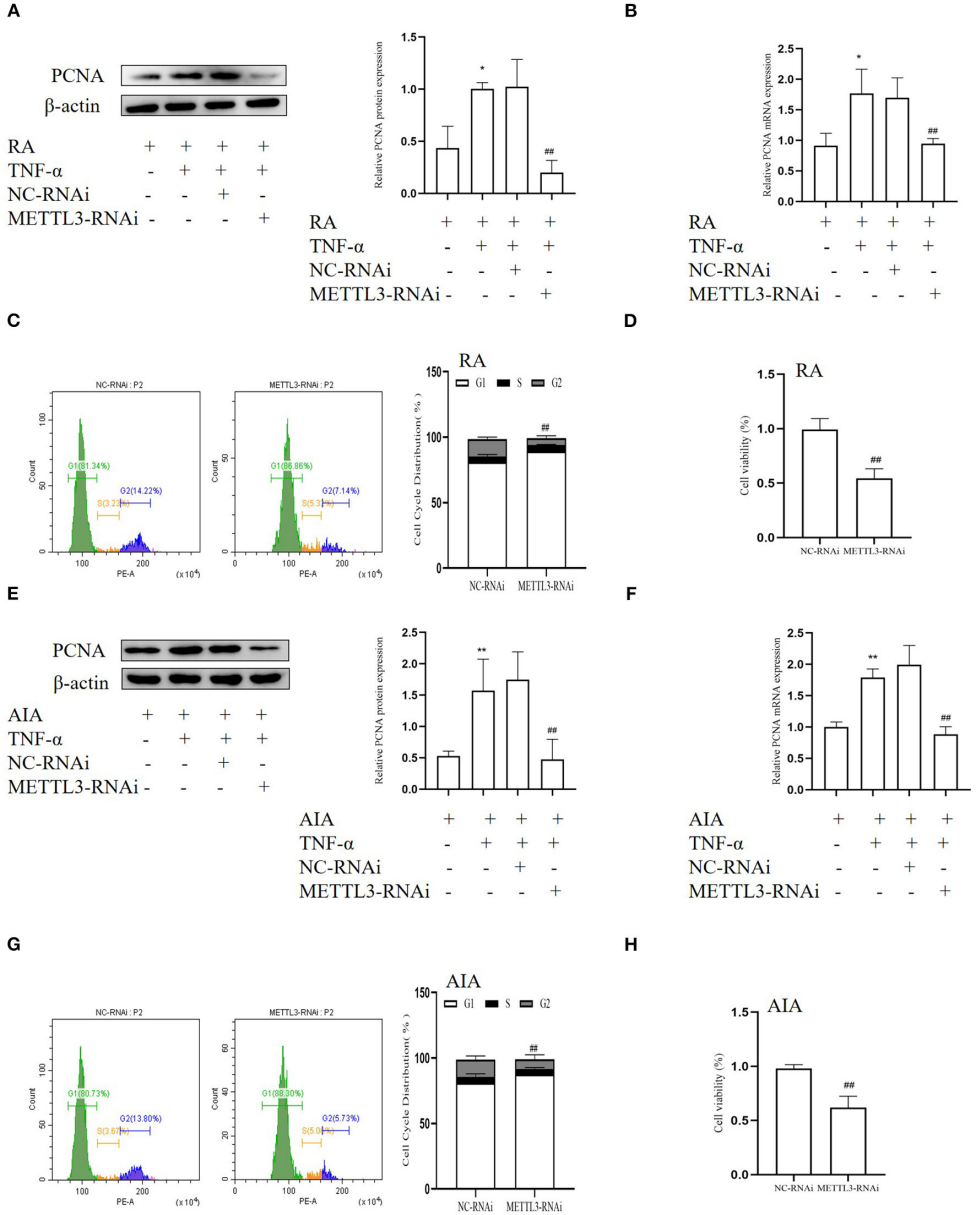
Methyltransferase-like 3 (METTL3) silencing inhibits cell proliferation in fibroblast-like synoviocytes (FLSs). **(A)** Proliferating cell nuclear antigen (PCNA) protein levels in TNF-α-incubated rheumatoid arthritis (RA)-FLSs after METTL3 knockdown, as detected by Western blotting. **(B)** PCNA mRNA level was detected by real-time quantitative PCR (qPCR) in TNF-α-incubated RA-FLSs after transfection with METTL3-RNAi. **(C)** Cell cycle analysis after incubating with METTL3-RNAi for 48 h, as assessed by fluorescence-activated cell sorting (FACS). **(D)** Cell growth of TNF-α-incubated RA-FLSs was measured by the 3-(4,5-dimethylthiazol-2-yl)-2,5-diphenyltetrazolium bromide (MTT) assay after transfection with METTL3-RNAi for 48 h. **(E)** PCNA protein level was detected by Western blotting in TNF-α-incubated adjuvant-induced arthritis (AIA)-FLSs after METTL3 silencing. **(F)** PCNA mRNA level was analyzed by real-time qPCR after transfection with METTL3-RNAi in TNF-α-incubated AIA-FLSs. **(G)** Cell cycle analysis was measured by FACS after transfection with METTL3-RNAi for 48 h. **(H)** Cell growth of TNF-α-incubated AIA-FLSs was analyzed by the MTT assay after transfection with METTL3-RNAi for 48 h. All of these values are presented as the mean ± SD. *NC*, negative control. *P < 0.05, **P < 0.01 vs. the control group. ^##^*P* < 0.01 vs. the NC group.

To research the variations following METTL3 knockdown, we used human-specific and rat-specific siRNAs to downregulate METTL3 expression, respectively. Western blotting, qPCR, MTT assays, and cell cycle assays were used. The Western blotting results (Figures 5A,E) showed that the protein level of the cell proliferation marker PCNA was decreased after transfection with METTL3 siRNA in TNF-α-incubated FLSs. Real-time qPCR results (Figures 5B,F) indicated that the PCNA mRNA level was markedly suppressed following METTL3 silencing. The MTT results (Figures 5D,H) showed that METTL3 knockdown significantly inhibited cell proliferation compared to the NC group. In addition, cell cycle analysis (Figures 5C,G) also showed that treatment with METTL3 siRNA in FLSs led to a decrease largely in the percentages of the S and the G2 phase and an increase in the percentages of the G0/G1 phases. These results suggested that METTL3 knockdown inhibits the cell proliferation of FLSs.

### Effect of METTL3 Overexpression Increases FLS Proliferation

**Figure 6:**
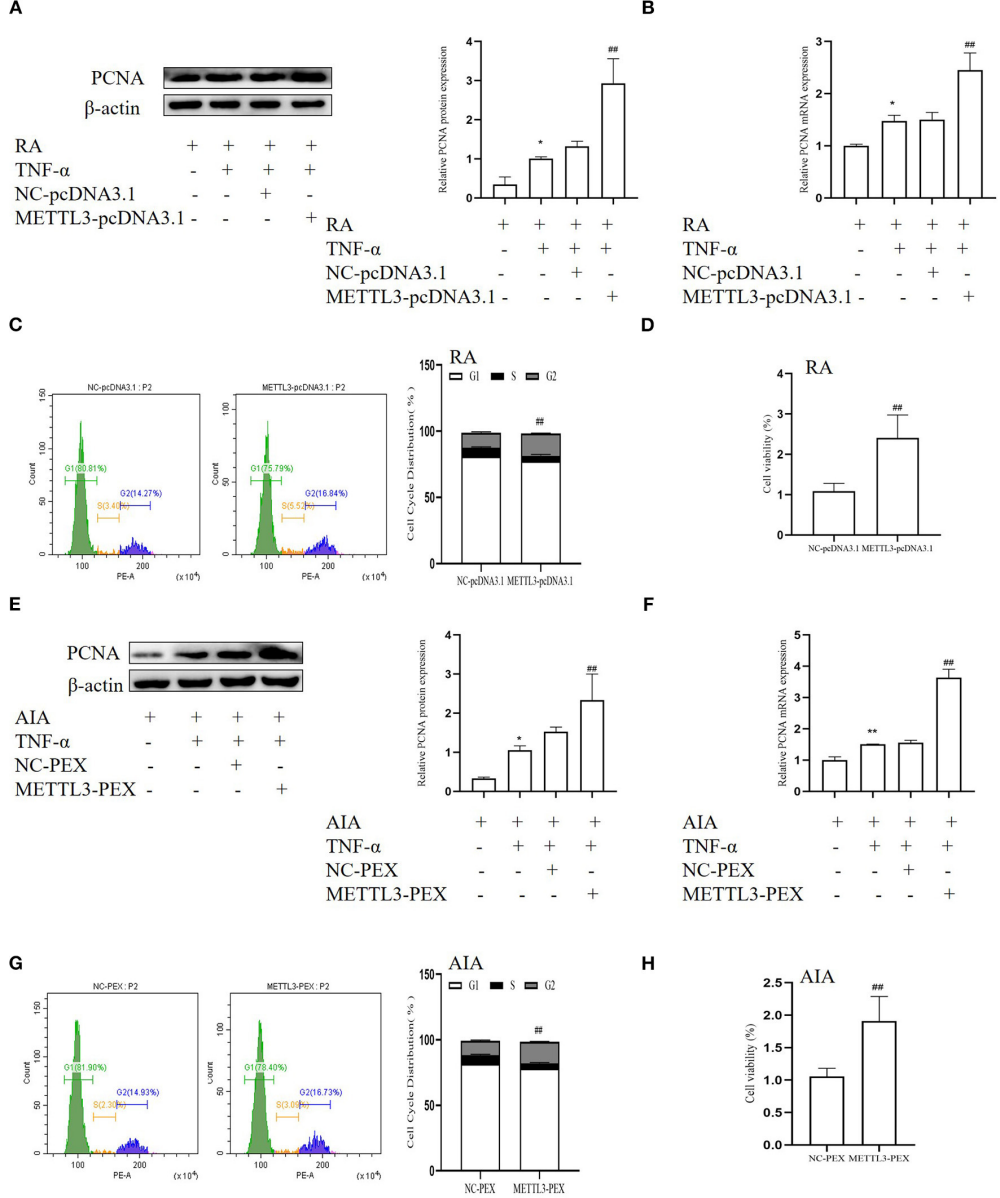
Effect of methyltransferase-like 3 (METTL3) overexpression increases fibroblast-like synoviocyte (FLS) proliferation. **(A)** Proliferating cell nuclear antigen (PCNA) protein levels in TNF-α-incubated rheumatoid arthritis (RA)-FLSs after METTL3-pcDNA3.1 transfection, as detected by Western blotting. **(B)** PCNA mRNA level was detected by real-time quantitative PCR (qPCR) in TNF-α-incubated RA-FLSs after transfection with METTL3-pcDNA3.1. **(C)** Cell cycle analysis after transfection with METTL3-pcDNA3.1 for 48 h, as detected by fluorescence-activated cell sorting (FACS). **(D)** Cell growth was measured by the 3-(4,5-dimethylthiazol-2-yl)-2,5-diphenyltetrazolium bromide (MTT) assay after transfection with METTL3-pcDNA3.1 for 48 h in TNF-α-incubated RA-FLSs. **(E)** PCNA protein levels in TNF-α-incubated adjuvant-induced arthritis (AIA)-FLSs after METTL3 overexpression, as detected by Western blotting. **(F)** PCNA mRNA level was analyzed by qPCR after transfection with METTL3-PEX in TNF-α-incubated AIA-FLSs. **(G)** Cell cycle analysis was measured by FACS after METTL3 overexpression for 48 h. **(H)** Cell growth was analyzed by the MTT assay after transfection with METTL3-PEX for 48 h in TNF-α-incubated AIA-FLSs. All of these values are presented as the mean ± SD. *NC*, negative control. *P < 0.05, **P < 0.01 vs. the control group. ^##^*P* < 0.01 vs. the NC group.

To further verify the function of METTL3 in FLS proliferation, we used the overexpression plasmids. The Western blotting (Figures 6A,E) and real-time qPCR (Figures 6B,F) results showed that the PCNA level in the METTL3 overexpression group was dramatically increased compared to the negative control group. The MTT results indicated that METTL3 overexpression promotes cell proliferation in RA-FLSs (Figure 6D), which also occurred in AIA-FLSs (Figure 6H). The cell cycle assay results (Figures 6C,G) indicated that METTL3 overexpression increased the percentages of RA-FLSs and AIA-FLSs in the S and G2 phases and decreased the percentages in the G0/G1 phases. These results all showed that METTL3 overexpression promotes cell proliferation.

### METTL3-RNAi Inhibits the Migration and Invasion in FLSs

**Figure 7:**
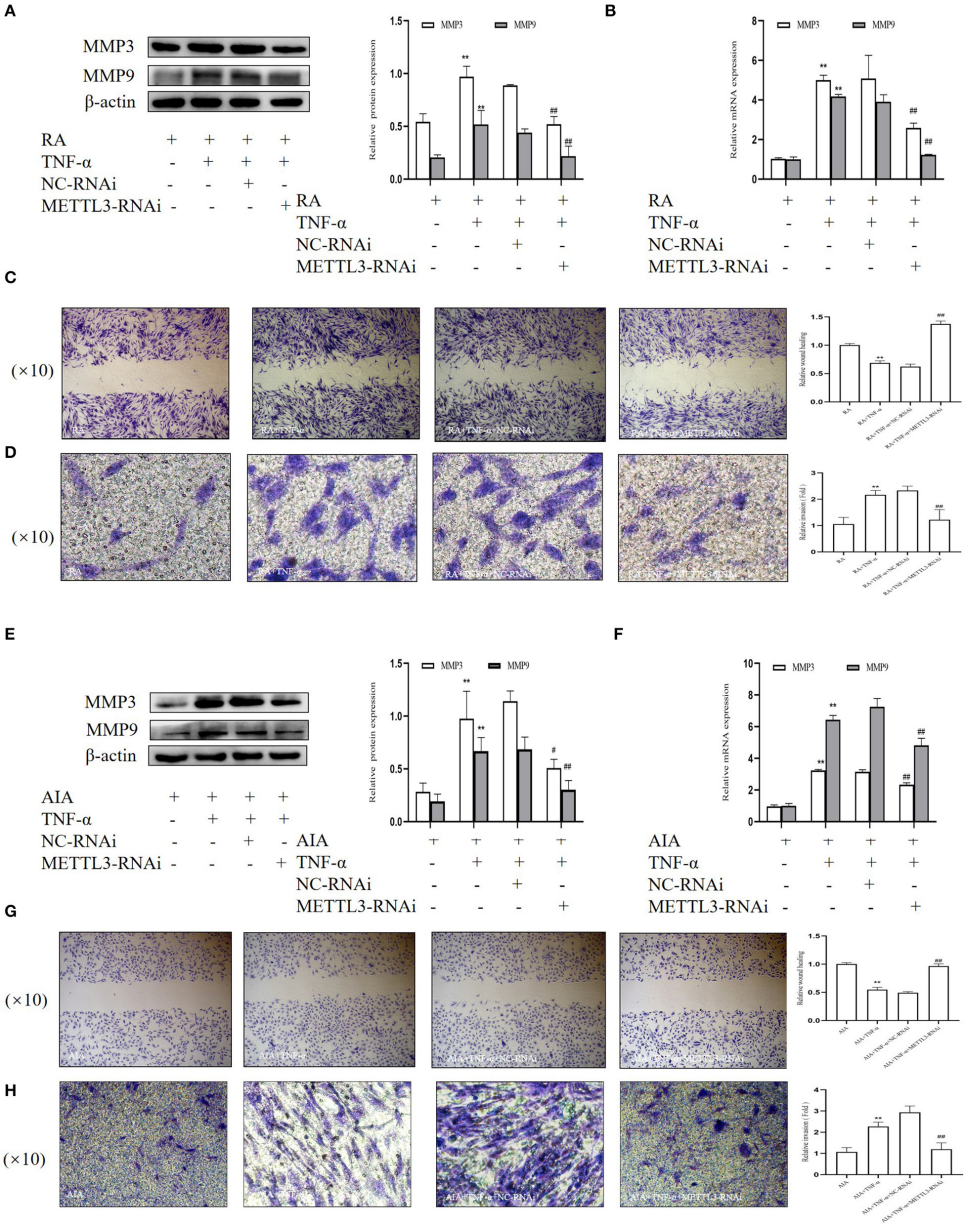
Methyltransferase-like 3 (METTL3)-RNAi inhibits migration and invasion in fibroblast-like synoviocytes (FLSs). **(A)** Western blotting was used to measure MMP3 and MMP9 protein levels after METTL3 silencing in rheumatoid arthritis (RA)-FLSs. **(B)** MMP3 and MMP9 mRNA levels were detected by real-time quantitative PCR (qPCR) in TNF-α-incubated RA-FLSs after transfection with METTL3-RNAi. **(C)** Representative photograph of TNF-α-treated, METTL3-knockdown RA-FLSs 24 h after wound formation (original magnification, ×10) and quantitative analysis of the wound healing extent. **(D)** After METTL3 silencing for 24 h in TNF-α-incubated RA-FLSs, Transwell invasion assay was photographed (original magnification, ×10), and quantitative analysis of the cell number. **(E)** Western blotting was used to measure MMP3 and MMP9 protein levels after METTL3 silencing in TNF-α-incubated adjuvant-induced arthritis (AIA)-FLSs. **(F)** qPCR was used to analyze MMP3 and MMP9 mRNA levels in TNF-α-incubated AIA-FLSs after transfection with METTL3-RNAi. **(G)** After METTL3 silencing in TNF-α-incubated AIA-FLSs, photographed after wound formation at 24 h (original magnification, ×10) and quantitative analysis of the wound healing extent. **(H)** After METTL3 silencing in TNF-α-incubated RA-FLSs, Transwell invasion assay was photographed (original magnification, ×10), and quantitative analysis of the cell number. All of these values are presented as the mean ± SD. *NC*, negative control. **P < 0.01 vs. the control group. ^#^*P* < 0.05, ^##^*P* < 0.01 vs. the NC group.

Having observed the influence of METTL3 on cell proliferation, we conducted a number of experiments to verify whether METTL3 knockdown affected RA-FLS and AIA-FLS migration and invasion. The Western blotting results (Figures 7A,E) suggested that the protein levels of MMP3 and MMP9 were obviously downregulated by METTL3 siRNA transfection. Additionally, the real-time qPCR results (Figures 7B,F) showed that the mRNA levels of MMP3 and MMP9 were significantly decreased in the METTL3-siRNA group. We also performed the wound healing assay and Matrigel-coated Transwell invasion assay. The wound healing assay results (Figures 7C,G) suggested that the migration ability of FLSs was decreased after METTL3 silencing. What is more, the Matrigel-coated Transwell invasion assay results (Figures 7D,H) showed that, compared to the negative control group, cell invasion was inhibited after transfection with METTL3-RNAi. These results all showed that METTL3 silencing in FLSs suppressed cell invasion and migration.

### Overexpression of METTL3 Promotes Migration and Invasion in RA- and AIA-FLSs

**Figure 8:**
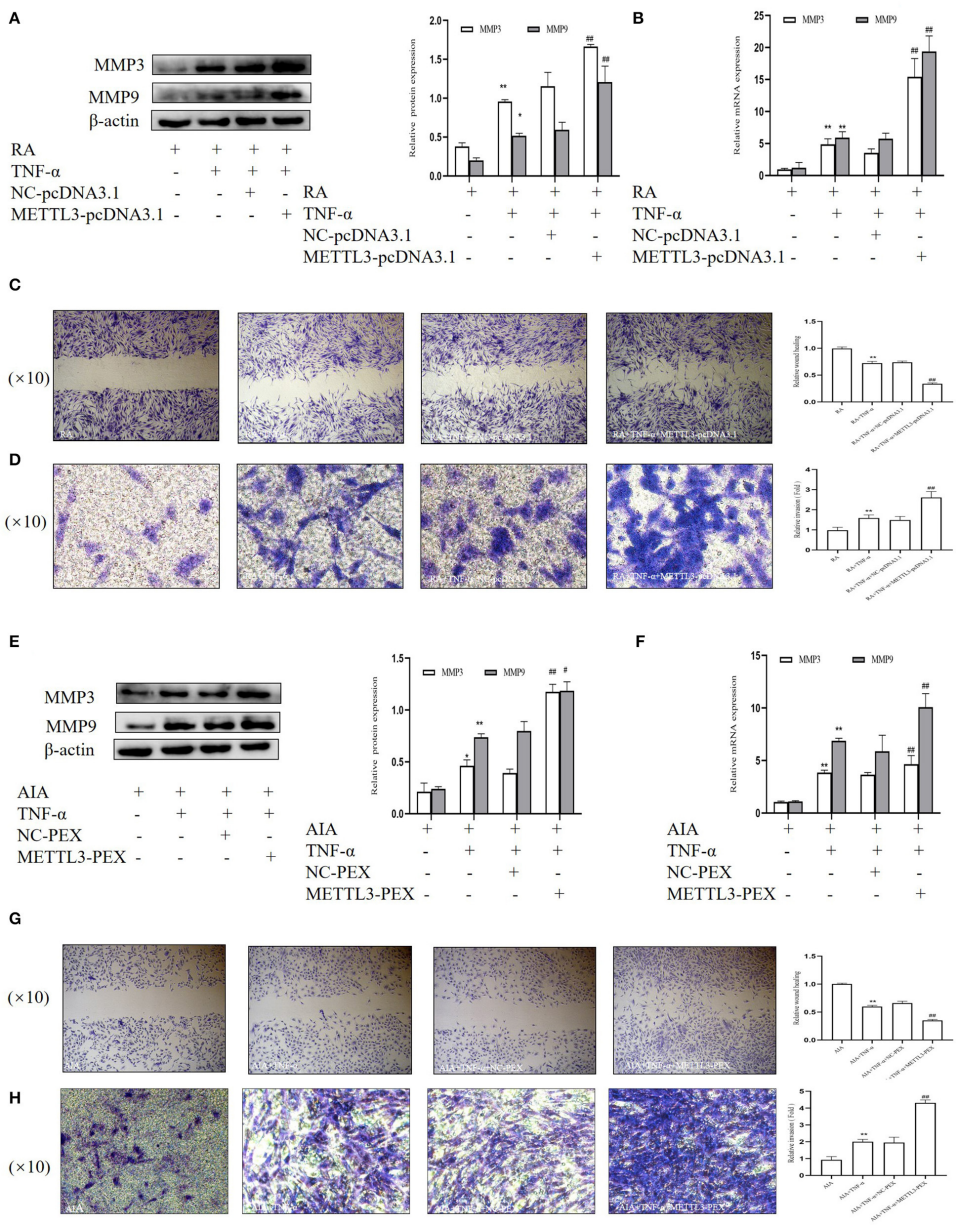
Overexpression of methyltransferase-like 3 (METTL3) promotes the migration and invasion in rheumatoid arthritis (RA) and adjuvant-induced arthritis (AIA) fibroblast-like synoviocytes (FLSs). **(A)** Western blotting was used to measure MMP3 and MMP9 protein levels after METTL3 overexpression in TNF-α-incubated RA-FLSs. **(B)** MMP3 and MMP9 mRNA levels were detected by real-time quantitative PCR (qPCR) in TNF-α-incubated RA-FLSs after transfection with METTL3-pcDNA3.1. **(C)** Representative photograph of TNF-α-treated METTL3-overexpressing RA-FLSs 24 h after wound formation (original magnification, ×10) and quantitative analysis of the wound healing extent. **(D)** After METTL3 overexpression at 24 h in TNF-α-incubated RA-FLSs, Transwell invasion assay was photographed (original magnification, ×10), and quantitative analysis of the cell number. **(E)** Western blotting was used to measure MMP3 and MMP9 protein levels after METTL3 silencing in TNF-α-incubated AIA-FLSs. **(F)** MMP3 and MMP9 mRNA levels were analyzed by qPCR in TNF-α-incubated AIA-FLSs after transfection with METTL3-PEX. **(G)** After METTL3 silencing in TNF-α-incubated AIA-FLSs, photographed after wound formation at 24 h (original magnification, ×10) and quantitative analysis of the wound healing extent. **(H)** After METTL3 overexpression at 24 h in TNF-α-incubated RA-FLSs, Transwell invasion assay was photographed (original magnification, ×10), and quantitative analysis of the cell number. All of these values are presented as the mean ± SD. *NC*, negative control. *P < 0.05, **P < 0.01 vs. the control group. ^#^*P* < 0.05, ^##^*P* < 0.01 vs. the NC group.

To further explore the invasion and migration in FLSs on the basis of METTL3 overexpression, we performed Western blotting, real-time qPCR, and wound healing and Matrigel-coated Transwell invasion assays. The Western blotting (Figures 8A,E) and real-time qPCR (Figures 8B,F) results showed that the MMP3 and MMP9 expression levels were significantly increased in the METTL3 overexpression group. The wound healing assay results (Figures 8C,G) indicated that the overexpression of METTL3 increased the migration ability of FLSs. The Matrigel-coated Transwell invasion assay was chosen, and the results (Figures 8D,H) suggested that FLS migration across the gel matrix into the lower chamber was promoted by METTL3 overexpression compared to the negative control group. Thus, these results all indicated that METTL3 overexpression promoted FLS migration ability and invasion ability.

### METTL3 Plays an Important Function in RA May Be *via* the NF-κB Signaling Pathway

**Figure 9:**
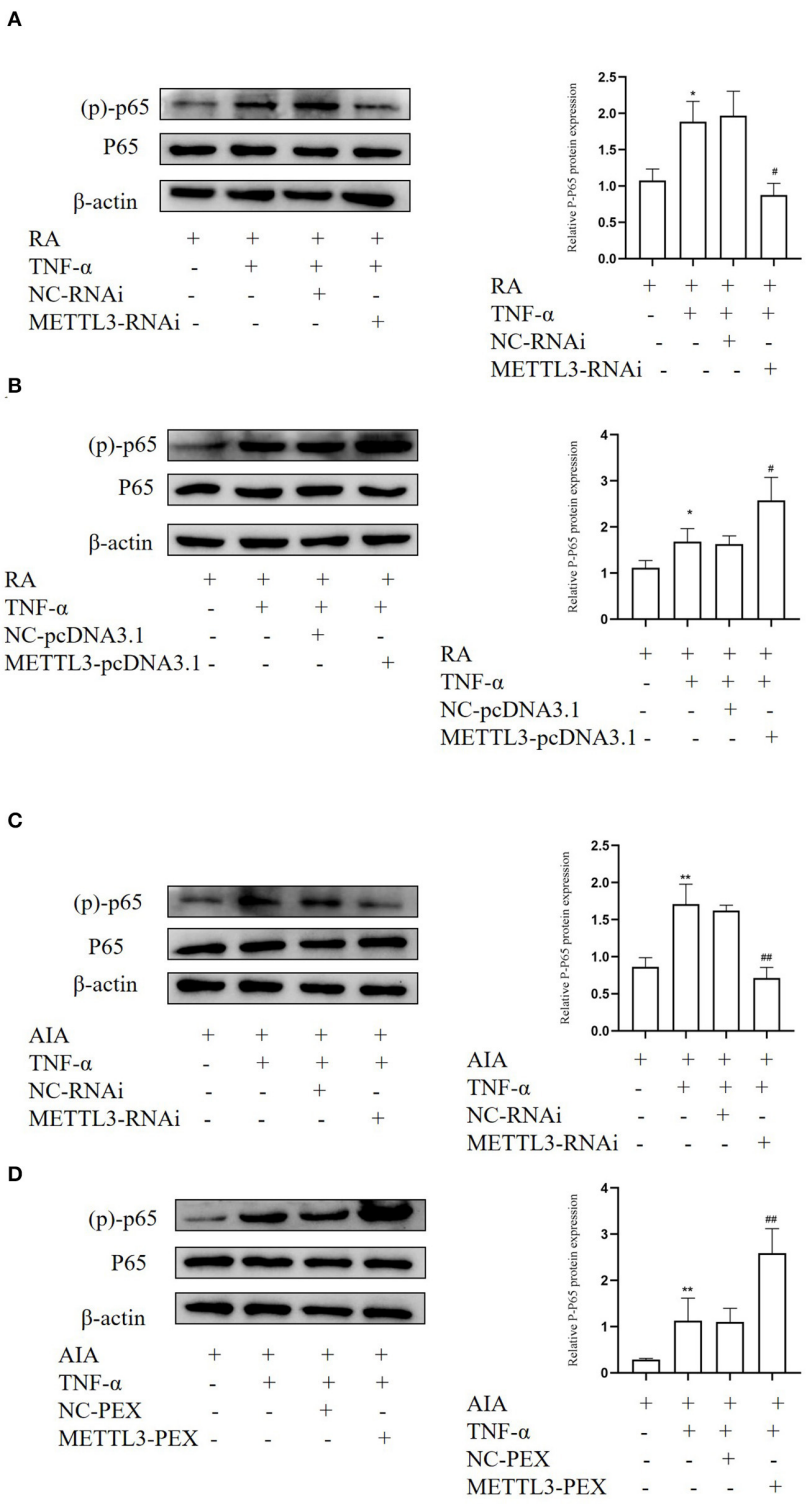
Methyltransferase-like 3 (METTL3) plays an important function in rheumatoid arthritis (RA) may be via the NF-κB signaling pathway. **(A)** Western blotting was used to measure (p)-p65 protein level in TNF-α-incubated RA-fibroblast-like synoviocytes (FLSs) after transfection with METTL3-RNAi. **(B**) Western blotting was used to detect (p)-p65 protein levels in TNF-α-incubated RA-FLSs after METTL3 overexpression. **(C)** Western blotting was used to detect (p)-p65 protein levels in TNF-α-incubated adjuvant-induced arthritis (AIA)-FLSs after METTL3 silencing. **(D)** Western blotting was used to measure (p)-p65 protein levels in TNF-α-incubated AIA-FLSs after METTL3 overexpression. All of these values are presented as the mean ± SD. *NC*, negative control. *P < 0.05, **P < 0.01 vs. the control group. ^#^*P* < 0.05, ^##^*P* < 0.01 vs. the NC group.

METTL3 expression is closely related to the NF-κB signaling pathway in multiple diseases. In order to study whether METTL3 regulates pro-inflammatory cytokine and FLS migration and invasion *via* the NF-κB signaling pathway, Western blotting was performed. We detected the level of (p)-p65 protein, which is a marker of NF-κB signaling pathway activation. The Western blotting results suggested that (p)-p65 was significantly downregulated after METTL3 silencing (Figures 9A,C) while upregulated after METTL3 overexpression (Figures 9B,D). These results revealed that METTL3 may affect the inflammatory response and FLS migration and invasion *via* the NF-κB signaling pathway.

## Discussion

Rheumatoid arthritis is an autoimmune disease with systemic manifestations. Local inflammatory cell infiltration into the joints leads to a chronic inflammatory reaction. The hyperplasia of the joint synovium leads to joint damage, joint deformities, obstacles, and loss of function, which seriously affect the patient's health and daily life (5, 29–31). At present, the exact pathological mechanism of rheumatoid arthritis remains unknown, and research on the mechanism is continuously being conducted. The AIA animal model is currently a commonly used animal model. It uses an injection of Freund's complete adjuvant into the toe. From the perspective of histology and immunology, the AIA animal model and RA are similar to a large extent (32). Therefore, in this study, we used the AIA animal model to explore the effect of METTL3.

Synovitis greatly promotes the development of RA. In synovitis, many fibroblasts proliferate and enter the synovial membrane, forming an inflammatory microenvironment and further damaging other tissues. FLSs are activated in the chronic inflammatory microenvironment and enhance the aggressiveness of RA (7, 33, 34). Therefore, FLSs are clearly important in the pathological mechanism underlying RA. Although a large number of studies have demonstrated the importance of FLSs, there are currently no highly effective treatments targeting FLSs, and the mechanisms underlying the effects of FLSs have not been explored. Therefore, targeted inhibition of FLS inflammation, proliferation, invasion, and migration remains a potential strategy for treating RA.

METTL3, the core component of the m^6^A methyltransferase complex, localized to nuclear speckles, is significantly altered in multiple malignancies and is closely related to poor prognosis (35). Numerous studies have shown that METTL3 takes part in a series of pathological states, including inflammation, cancer, and immune regulation (36–38). In this study, we investigated whether METTL3 affects the inflammatory response and cell proliferation, invasion, and migration. We used immunohistochemical staining, Western blotting, and real-time qPCR to assess METTL3 expression. It is worth noting that METTL3 was obviously higher in RA and AIA synovial tissues than in the control group synovial tissues. However, the number of samples involved in our study was relatively small, and we aim to collect more samples in future research to improve our experimental data.

The RA inflammatory microenvironment involves many pro-inflammatory factors and chemokines, among which TNF-α is one of the main pro-inflammatory cytokines (9). Research has shown that treatment with 10 ng/ml TNF-α can stimulate FLS activation and promote inflammatory cytokine production (39), altering the expression levels of multiple genes in FLSs (9). We extracted and identified primary fibroblast-like synovial cells, stimulated them with 10 ng/ml TNF-α *in vitro*, and used Western blotting and qPCR to explore the expression of METTL3. The results indicated that METTL3 expression was upregulated.

FLSs exhibit tumor-like characteristics (7). Excessive proliferation, migration, and invasion are important behavioral characteristics of malignant cells. Further experimental results suggested that, after METTL3 silencing, the cell cycle became arrested in the G0/G1 phases, while the wound healing and Transwell invasion assays indicated that silencing METTL3 suppressed the proliferation, migration, and invasion in RA-FLSs and AIA-FLSs. Additionally, the levels of PCNA (an indicator of proliferation) and MMP3 and MMP9 (related to cell migration and invasion) were decreased by METTL3 knockdown. After obtaining these experimental results, we selected METTL3 overexpression plasmids to observe whether METTL3 overexpression facilitated the proliferation, invasion, and migration in FLSs. To our surprise, METTL3 overexpression increased the proliferation, invasion, and migration in FLSs. At the same time, we found that with silencing METTL3, the levels of IL-6, IL-1β, and TNF-α were decreased obviously, while METTL3 overexpression significantly increased them.

Recent studies have pointed out that the regulatory mechanisms of METTL3 in diseases are related to the NF-κB signaling pathway. The NF-κB signaling pathway, as a classic inflammation pathway, is activated in RA, and it is closely related to RA progression because it upregulates MMPs. At the same time, studies have indicated that cytokines activate the NF-κB signaling pathway, which then further upregulates cytokine expression, which in turn affects cell proliferation and migration. In this study, METTL3 knockdown decreased the expression of (p)-p65 in FLSs, while the overexpression of METTL3 increased the expression of (p)-p65 in FLSs.

In summary, based on all the above experimental results, we believe that METTL3 not only mediates FLS pro-inflammatory cytokine expression in FLSs but also participates in FLS proliferation, invasion, and migration. This may be related to the activation of the NF-κB signaling pathway. However, there are still limitations in our experiment, and the in-depth mechanisms underlying the effects of METTL3 on FLS proliferation, invasion, and migration remain unclear. In conclusion, we demonstrated that METTL3 expression in synovial tissues was significantly increased. Silencing METTL3 inhibits the production of pro-inflammatory cytokines and the ability of FLSs to proliferate, migrate, and invade, while overexpressing METTL3 promotes these factors. Our findings indicated that METTL3 may play a key role in RA progression by activating the NF-κB signaling pathway. Manipulating METTL3 levels may be a new RA treatment approach.

## Conclusion

The results of this study show that: (1) METTL3 is upregulated obviously in human RA synovial tissues and rat AIA synovial tissues; (2) METTL3-RNAi inhibited the expression of pro-inflammatory factors in RA-FLSs and AIA-FLSs, as well as the proliferation, invasion, and migration of these cells; (3) in RA-FLSs and AIA-FLSs, METTL3 overexpression promoted pro-inflammatory cytokine expression, proliferation, invasion, and migration; and (4) METTL3 expression may be closely related to the NF-κB signaling pathway.

## Data Availability Statement

The original contributions presented in the study are included in the article/Supplementary Material, further inquiries can be directed to the corresponding author/s.

## Ethics Statement

The animal study was reviewed and approved by Anhui Medical University for the Care and Use of Laboratory.

## Author Contributions

WS performed all experiments, analyzed the data, and wrote the manuscript. YZha helped to collect the RA and OA synovial tissues. SL and YZhe helped to isolated FLSs and helped to Western blot, Q-PCR and histological analysis. XL and CH participated in the design of the study. XM helped to revise the manuscript. JL conceived the study and revised the manuscript. All authors approved the final manuscript.

## Conflict of Interest

The authors declare that the research was conducted in the absence of any commercial or financial relationships that could be construed as a potential conflict of interest.
