# Supplementary figures and images for: METTL3 Promotes Activation and Inflammation of FLSs Through the NF-κB Signaling Pathway in Rheumatoid Arthritis
Source: Front Med (Lausanne). 2021 Jul 6;8:607585. doi: 10.3389/fmed.2021.607585 (PMC8290917; doi:10.3389/fmed.2021.607585)

Supplementary file

| Disease | RA (n=5)    | OA (n=3)   |
|---------|-------------|------------|
| CRP     | 12.68±4.29  | 1.27±0.49  |
| ESR     | 56.2±22.63  | 10.33±3.79 |
| RF      | 90.22±18.13 | 6.07±2.65  |

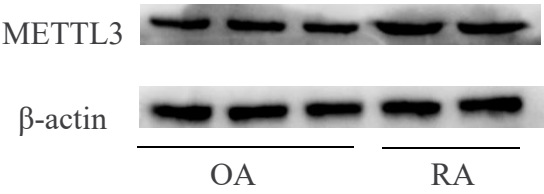

Supplement: Supplementary file 1 [file Data_Sheet_1.PDF]
